# Testicular neuroendocrine tumor in a 32‐year‐old man: A case report

**DOI:** 10.1002/ccr3.8620

**Published:** 2024-03-04

**Authors:** Reza Dehghaniathar, Asaad Moradi, Nikoo Emtiazi

**Affiliations:** ^1^ Department of Urology, Firoozgar Hospital, School of Medicine Iran University of Medical Sciences Tehran Iran; ^2^ Department of Pathology, Firoozgar Hospital, School of Medicine Iran University of Medical Sciences Tehran Iran

**Keywords:** carcinoid tumor, neuroendocrine tumors, testicular cancer, testicular neuroendocrine tumor

## Abstract

**Key Clinical Message:**

A 32‐year‐old male with painful scrotal swelling who underwent radical orchiectomy and was diagnosed with a testicular neuroendocrine tumor. Determining whether testicular neuroendocrine tumor is primary or metastasis from another origin is crucial.

**Abstract:**

Testicular neuroendocrine tumors (TNET) are one of the rarest human neoplasms, with about 132 identified cases until 2015. Testicular neuroendocrine tumors are frequently manifest with painless scrotal swelling or mass. In this study, we present a 32‐year‐old male with a chief complaint of painful progressive swelling of the right testicle without any history of trauma. All laboratory tests were within the normal range. Ultrasound revealed two hyper‐vascular masses in the right testicle. Computed tomography was performed, and patients had no evidence of metastases. The patient underwent right radical orchiectomy, and a histopathological examination diagnosed the specimen with a well‐differentiated testicular neuroendocrine tumor. Because of the rarity of TNET, there are many controversial issues in the treatment, especially in cases with metastatic TNET. Determining whether testicular neuroendocrine tumor is primary or metastasis from another origin is crucial. Further studies are required to achieve optimum treatment for TNET.

## 1 INTRODUCTION

Testicular cancer is one of the infrequent malignancies with incidence of about 1% among male individuals. Around 95% of testicular cancers arise from germ cell tumors, the most frequent type.^1^ Further, the testicle is an infrequent location for developing neuroendocrine tumors, as less than 1% of testicular tumors are identified to have a neuroendocrine nature. In contrast, most neuroendocrine tumors are found in the gastrointestinal tract. So, testicular neuroendocrine tumors (TNET) are one of the rarest neoplasms, with about 132 identified cases until 2015.^2^

Similar to other testicular neoplasms, TNETs are frequently manifested with painless scrotal swelling or mass, while about 10% of reported cases had carcinoid syndrome, which is characterized by flushing, chronic diarrhea, abdominal pain, bronchospasm, and sweats.^2^, ^3^ The median age at the time of diagnosis is 38 years, serum biomarkers including β‐gonadotrophin chorionic humaine, α‐feto protein, and lactate dehydrogenase are usually not elevated, and about 6% of TNETs are metastatic at the time of diagnosis.^2^ In this study, we aim to present a case of TNET and highlight the diagnosis and management process.

## 2 CASE HISTORY

A 32‐year‐old male presented to the urology Department of the Firoozgar Hospital (Tehran, Iran) with a two‐month history of painful progressive swelling of the right testicle. The patient denied any history of trauma or previous scrotal surgeries. His past medical and familial history was not notable.

### 2.1 Physical examination and laboratory tests

Physical examination revealed an enlarged right testicle and tenderness on palpation. The epididymal demarcation was distinct, and no inguinal lymphadenopathy was identified. Also, laboratory tests, including complete blood count, liver function tests, renal function tests, and tumor markers including α‐fetoprotein (2.06 ng/mL), beta‐human chorionic gonadotropin (0.1 IU/L), and lactate dehydrogenase (376 U/L) were within normal limits.

### 2.2 Differential diagnoses, investigations, and treatment

**FIGURE 1:**
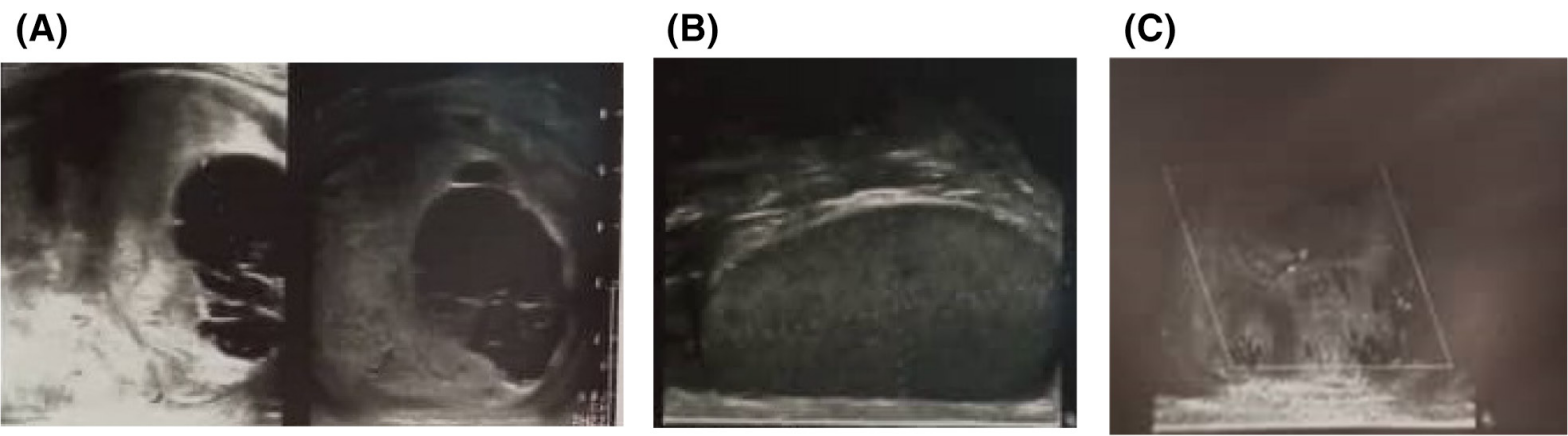
Testicular ultrasound: (A) solid heterogenic mass within the inferior part of the right testicle (diameter: 36 × 44 × 32 mm), (B) moderate hydrocele in the right hemi‐scotoma, (C) another solid heterogenic mass (diameter: 63 × 49 × 45 mm) at the upper portion of the right testicle.

As shown in Figure 1, scrotal 10 MHz ultrasound revealed a solid hyperechoic mass within the inferior part of the right testicle (diameter: 36 × 44 × 32 mm) and another solid cystic mass (diameter: 49 × 45 × 63 mm) at the upper portion of the right testicle. Also, in the Doppler examination, both masses were hyper‐vascular. Also, a moderate hydrocele was identified in the right hemi‐scotoma. A chest, abdomen, and pelvic computed tomography (CT) scan and bone scintigraphy were performed after the operation to assess the possible metastases, and no evidence of metastasis was identified. Also, the patient underwent an endoscopy and colonoscopy to explore the gastrointestinal tumor; however, it was negative.

Based on the ultrasound results, the patient underwent right radical orchiectomy, and the specimens, including spermatic cord (3.5 × 3 cm), tumors, epididymis (15 × 1 cm), and right testicle (12.5 × 7 × 7 cm), were sent for histopathological examinations. A well‐defined homogeneous cream‐yellow rubbery mass (diameter: 6 × 5.5 × 5 cm) with a cystic attached area to the lateral portion (35 mm in greatest diameter) was identified. Tunica vaginalis, epididymis, spermatic cord, and surgical margin were tumors‐free. In the testicular tissue, the islands of cells for mainly small acini and cords or mainly sheets separated by fibrous stroma were identified. The cells had granular eosinophilic cytoplasm round nuclei with granular chromatin, and no significant mitotic activity or necrosis was identified.

**FIGURE 2:**
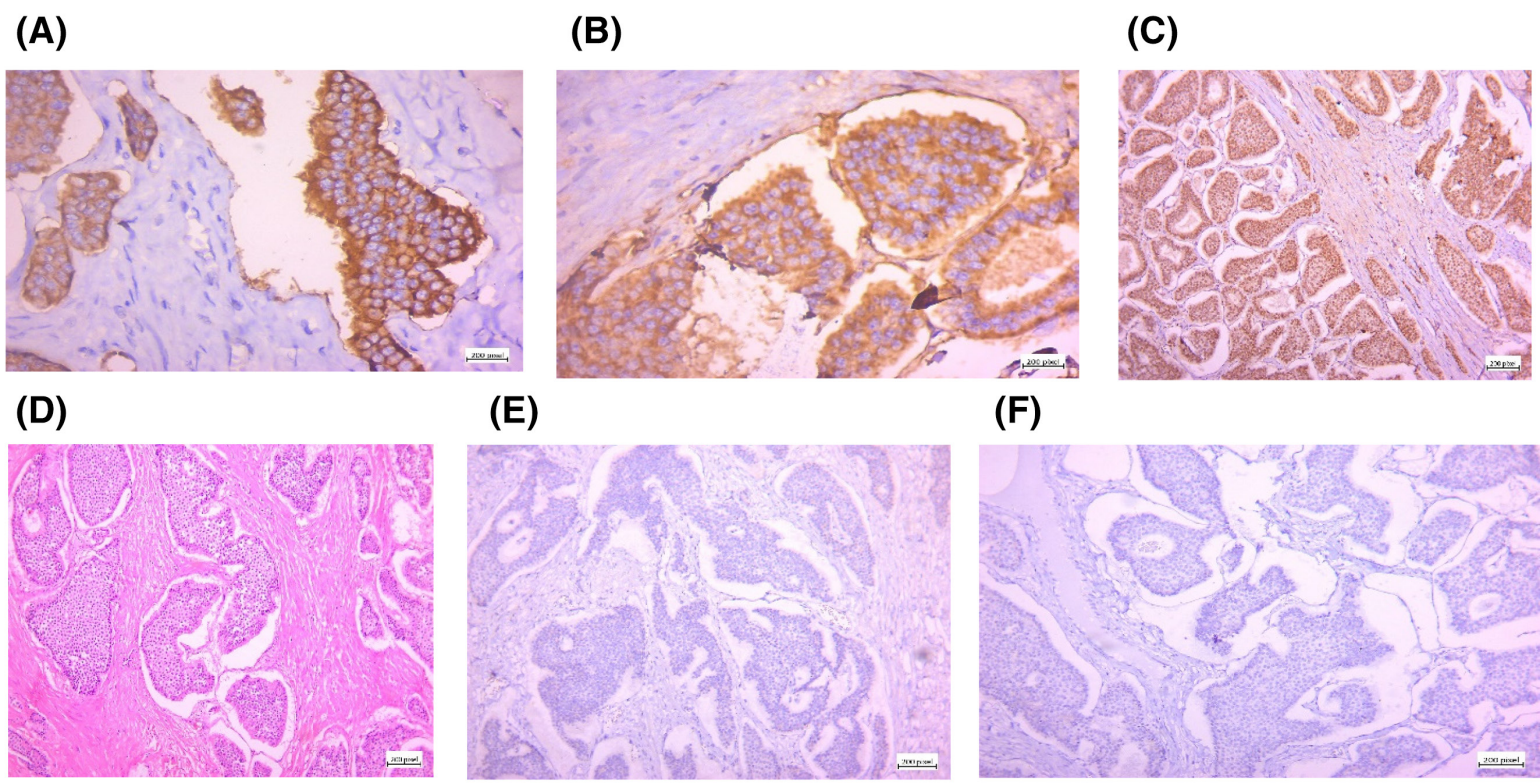
(A) Synaptophysin immunomarker shows diffuse cytoplasmic staining for synaptophysin (×40). (B) Chromogranin A show disuse granular staining. (C) Insulinoma‐associated protein 1 (INSM 1) show diffuse nuclear staining (×10). (D) Organoid architecture: tumor cells arranged in nests, trabecular or insular pattern cells are Monotonous regular with round or oval nuclei with salt and pepper chromatin and moderate eosinophilic granular cytoplasm, (hematoxylin and eosin ×10). (E) Negative reactivity for ttf1 (×10). (F) Negative reactivity for cdx2 (×10).

Immunohistochemistry staining showed negative reactivity for SALL4, CD117, CDX2 and TTF1. In contrast, positive reactivity for Plap, CD56, INSM1, and Synaptophysin were identified. KI67 was estimated to be up to 2%. Also, Chromogranin showed weak positive reactivity. As shown in Figure 2, the histopathological examination revealed a well‐differentiated neuroendocrine tumor (mono‐dermal teratoma).

### 2.3 Outcome and follow‐up

During 1 year of follow‐up, no distinct metastasis was identified, and the patient had no post‐surgical complications.

## 3 DISCUSSION

TNET was first introduced by Simon et al. in 1954.^4^ However, TNETs are known as carcinoid tumors. TNET is extremely rare, with a mean prevalence of less than 1% among testicular tumors. According to the latest World Health Organization classification for testicular tumors, TNET is a subtype of germ cell tumors unrelated to germ cell neoplasia.^5^

The mean age at the diagnosis is 39 years, ranging from 10 to 83 years. The most common symptom is the painless scrotal mass; however, about 17% of patients present with scrotal pain. About 10% of patients are diagnosed with carcinoid syndrome; however, it is uncommon. Serum tumor markers such as β‐gonadotrophin chorionic Hormone, α‐feto protein, and lactate dehydrogenase are within the normal range as in our patient. Among TNETs, primary TNET is the most common one; however, about 7% of TNETs are metastatic tumors of another origin. Also, about 16% of TNETs are associated with testicular teratoma.^2^, ^6^

Same as other testicular tumors, ultrasound is an available and accurate modality for pre‐operative evaluation. However, abdominopelvic magnetic resonance imaging (MRI) and CT‐scan can be used to find other sites metastasis. As a testicular mass, the standard treatment for the TNETs is orchiectomy. In cases of lymph node metastasis, patients should undergo retroperitoneal lymph node dissection. Patients with metastatic TNETs should undergo combination therapy with streptozocin, 5‐fluorouracil, and cyclophosphamide (or doxorubicin) as the standard chemotherapy protocol. However, some studies suggest chemotherapy with cisplatin, etoposide, ifosfamide, and epirubicin in lymph node or other site metastasis cases.^2^, ^7^, ^8^, ^9^, ^10^, ^11^

In conclusion, because of the rarity of the TNET, there are many controversial issues in the treatment, especially in cases with metastatic TNET. Determining whether TNET is primary or metastasis from another origin is crucial. Further studies are required to achieve optimum treatment for TNETs.

## AUTHOR CONTRIBUTIONS

**Reza Dehghaniathar:** Conceptualization; writing – review and editing. **Asaad Moradi:** Writing – original draft. **Nikoo Emtiazi:** Visualization; writing – review and editing.

## FUNDING INFORMATION

All authors declare that they did not receive funding for this study.

## CONFLICT OF INTEREST STATEMENT

All authors declare they have no financial or non‐financial competing interests.

## ETHICS STATEMENT

The Iran University of Medical Sciences Ethics Committee approved this study. Also, informed consent was obtained from the patient to participate in this study.

## CONSENT

Informed consent was obtained from the patient to publish figures and information.

## Data Availability

More information about the patient is available by contacting the corresponding author.
